# Regulation of CyR61 expression and release by 3-mercaptopyruvate sulfurtransferase in colon cancer cells

**DOI:** 10.1016/j.redox.2022.102466

**Published:** 2022-09-08

**Authors:** Kelly Ascenção, Bassma Lheimeur, Csaba Szabo

**Affiliations:** Chair of Pharmacology, Faculty of Science and Medicine, University of Fribourg, Fribourg, Switzerland

**Keywords:** Hydrogen sulfide, CyR61, Apoptosis, S1PR, p38MAPK, Colon cancer

## Abstract

Cysteine-rich angiogenic inducer 61 (CYR61, also termed CCN family member 1 or CCN1), is a matricellular protein encoded by the CYR61 gene. This protein has been implicated in the regulation of various cancer-associated processes including tumor growth, angiogenesis, tumor cell adhesion, migration, and invasion as well as the regulation of anticancer drug resistance. Hydrogen sulfide (H_2_S) is a gaseous endogenous biological mediator, involved in the regulation of cellular bioenergetics, angiogenesis, invasion, and chemotherapeutic resistance in several types of cancer. H_2_S is produced by three enzymes: cystathionine-β-synthase (CBS), cystathionine-γ-lyase (CSE) and 3-mercaptopyruvate sulfurtransferase (3-MST). The current studies were set up to investigate if CBS or 3-MST regulates CyR61 in colon cancer cells in the context of the regulation of proliferation, migration, and survival. The study mainly utilized HCT116 cells, in which two of the principal H_2_S-producing enzymes, CBS and 3-MST, are highly expressed. The H_2_S donor GYY4137 and the polysulfide donor Na_2_S_3_ activated the CyR61 promoter in a concentration-dependent fashion. Aminooxyacetic acid (AOAA), a pharmacological inhibitor of CBS as well as HMPSNE: 2-[(4-hydroxy-6- methylpyrimidin-2-yl)sulfanyl]-1-(naphthalen-1-yl)ethan-1-one, a pharmacological inhibitor of 3-MST inhibited CyR61 mRNA expression. This effect was more pronounced in response to HMPSNE than to AOAA and occurred through the modulation of S1PR via ATF1 and CREB. CyR61 was found to play an active, but relatively minor role in maintaining colon cell proliferation. HMPSNE markedly suppressed the secretion/release of CyR61 from the colon cancer cells. Moreover, HMPSNE promoted colon cancer cell apoptosis; endogenously produced CyR61 was found to counteract this effect, at least in part via RhoA activation. Taken together, we conclude that the upregulation of 3-MST in cancer cells exerts cytoprotective effects and confers the cancer cells a more aggressive phenotype – at least in part via the modulation of CyR61 expression and release.

## List of abbreviations:

3-MST: 3-mercaptopyruvate sulfurtransferase
ACLY: ATP citrate lyase
AP-1: activating protein-1
APC: allophycocyanin
AOAA: aminooxyacetic acid
ATCC: American Type Culture Collection
ATF: Activating Transcription Factor
BID: BH3 interacting-domain death agonist
BSA: bovine serum albumin
CAV1: caveolin-1
CBS: cystathionine-β-synthase
cDNA: complementary deoxyribonucleic acid
CRC: colorectal cancer
CREB: cAMP-response element binding protein
CSE: cystathionine-γ-lyase
CyR61: cysteine-rich angiogenic inducer 61
ELISA: enzyme-linked immunosorbent assay
FACS: fluorescence-activated cell sorting
FBS: fetal bovine serum
GAPDH: glyceraldehyde 3-phosphate dehydrogenase
GYY4137: 4-methoxyphenyl(morpholino)phosphinodithioate morpholinium salt
H_2_S: hydrogen sulfide
HIF-1α: hypoxia-inducible factor-1α
HMPSNE: 2-[(4-hydroxy-6-methylpyrimidin-2-yl)sulfanyl]-1-(naphthalen-1-yl)ethan-1-one
HRP: horseradish peroxidase
IgG: immunoglobulin G
JUN: jun proto-oncogene
MFI: mean fluorescence intensity
MET: mesenchymal-to-epithelial transition
Na_2_S_3_: disodium trisulfide
p38MAPK: p38 mitogen-activated protein kinase
PBS: phosphate-buffered saline
PI: propidium iodide
PCR: polymerase chain reaction
PDVF: polyvinylidene fluoride
PTEN: phosphatase and tensin homolog
qPCR: quantitative polymerase chain reaction
qRT-PCR: real-time reverse transcription-PCR
RhoA: ras homolog family member A
RIPA: radioimmunoprecipitation assay
RNA: ribonucleic acid
RT: reverse transcriptase
S1PR: sphingosine-1-phosphate receptor
SDS: sodium dodecyl sulfate
SEM: standard error of the mean
Shh: sonic hedgehog
Sp1: specificity protein 1
TBST: mixture of tris-buffered saline and Tween 20

## 1 Introduction

Hydrogen sulfide (H_2_S) is a mammalian gasotransmitter, generated in various cell types in a regulated fashion by 3 principal enzymes: cystathionine-γ-lyase (CSE), cystathionine-β-synthase (CBS), and 3-mercaptopyruvate sulfurtransferase (3-MST) [[1], [2], [3], [4]]. Upregulation of various H_2_S-producing enzymes has been demonstrated in various cancer cells over the past decade. H_2_S in cancer cells promotes cell proliferation and migration, stimulates cellular bioenergetics, enhances angiogenesis, promotes cancer cell dedifferentiation, invasion, and metastasis, and confers resistance to chemotherapeutic agents and to ionizing radiation [[4], [5], [6], [7], [8], [9], [10], [11], [12], [13], [14], [15]]. Aminooxyacetic acid (AOAA), an inhibitor of PLP-dependent enzymes, is commonly used to inhibit CBS *in vitro* and *in vivo* [1,16,17]. For 3-MST, HMPSNE (2-[(4-hydroxy-6-methylpyrimidin-2-yl)sulfanyl]-1-(naphthalen-1-yl)ethan-1-one) is a commonly used competitive inhibitor of this enzyme [11,[18], [19], [20], [21]]. Pharmacological inhibition of H_2_S enzymes in various types of cancer cells – including colon cancer, ovarian cancer, breast cancer and others – decreases proliferation, migration, and cellular bioenergetics, induces cancer cells apoptosis, sensitizes cancer cells to chemotherapeutic drug and induces mesenchymal-to-epithelial transition [[5], [6], [7], [8], [9], [10], [11], [12], [13], [14], [15],[19], [20], [21], [22], [23], [24], [25]].

Cysteine-rich angiogenic inducer 61 (CYR61 or CCN family member 1) is a cell-associated as well as secreted matricellular protein involved in tumor formation, growth, vascularization, angiogenesis, adhesion, drug resistance, migration, and invasion [[26], [27], [28], [29], [30], [31], [32], [33], [34], [35], [36], [37], [38], [39], [40]]. In colorectal cancer, CyR61 has been reported to promote cell migration, invasion, and metastasis; high expression of this protein was shown to correlate with poor prognosis [26,[41], [42], [43], [44]]. The CyR61 promoter, which is responsive to the transcription factor Sp1 (specificity protein 1), has been shown to be activated by various factors that are relevant for the pathogenesis of cancer, such as hypoxia-inducible factor-1α, cAMP response element binding protein (CREB) and activator protein-1 (AP-1) [[45], [46], [47]]. Sphingosine 1-phosphate (S1P) induces CyR61 through the induction of RhoA GTPase and p38MAPK signaling pathways, by activating the CREB and AP-1 regions of the CyR61 promoter [[45], [46], [47], [48], [49]].

The Wnt/β-catenin pathway plays a fundamental role in cancer development and metastasis [50,51]. Several studies have demonstrated that this pathway is both interlinked with CyR61 and with various H_2_S-associated pathways [20,[52], [53], [54]]. Thus, here we have examined if CyR61 is regulated by endogenous H_2_S in cancer. We have used HCT116 cells, a human colon cancer cell line *in vitro*, where the importance of both CyR61 and the H_2_S producing enzymes CBS and 3-MST have previously been demonstrated.

## 2 Results

**Fig. 1:**
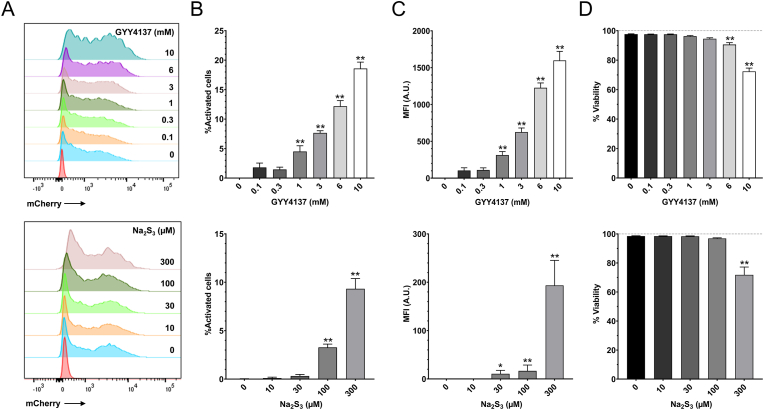
**H**_**2**_**S and polysulfides activate the CyR61 promoter.** Flow-cytometric analysis of mCherry protein after treatment of HCT116 cells with increasing concentrations of GYY4137 or Na_2_S_3_ for 48 h, mCherry expression reflects the activation of the CyR61 promoter. **A**: Histogram showing the increase of the mCherry positive cell population in the presence of increasing concentrations of GYY4137 or Na_2_S_3_. **B**: Percentage of activated cells compared to control. Data are shown as mean ± SEM, n = 4, **p < 0.01 compared to control. **C**: mCherry mean fluorescence intensity (MFI) compared to control. Data are shown as mean ± SEM, n = 4, **p < 0.01 compared to control. **D**: Cell viability after 48 h treatment with increasing concentrations of GYY4137 or Na_2_S_3_. Data are shown as mean ± SEM, n = 4, **p<0.01 compared to control.

**H**_**2**_**S and polysulfides activate the CyR61 promoter.** To examine the effect of H_2_S and polysulfides on the activity of the CyR61 promoter, we have introduced a viral vector containing a CyR61 promoter with an inducible mCherry reporter into HCT116 cells. HCT116 is a human colon cancer cell line, in which the pathogenetic role of CBS and 3-MST has previously been demonstrated [4,[9], [10], [11],^20^]. The slow-release H_2_S generator GYY4137, as well as the polysulfide donor Na_2_S_3_ concentration-dependently activated the CyR61 promoter (Fig. 1A,B,C). In line with the bell-shaped concentration-response character of H_2_S – where low and intermediate concentrations of this mediator serve regulatory roles, while higher concentrations become inhibitory, at least in part via inhibition of mitochondrial Complex IV [1] – a slight cytotoxic effect was observed with the highest GYY4137 and Na_2_S_3_ concentrations used (Fig. 1D). However significant CyR61 promoter activation was already noted at lower concentrations of GYY4137 (1–3 mM) and Na_2_S_3_ (100 μM), where these agents exerted no adverse effects on cell viability (Fig. 1A,B,C).

**Fig. 2:**
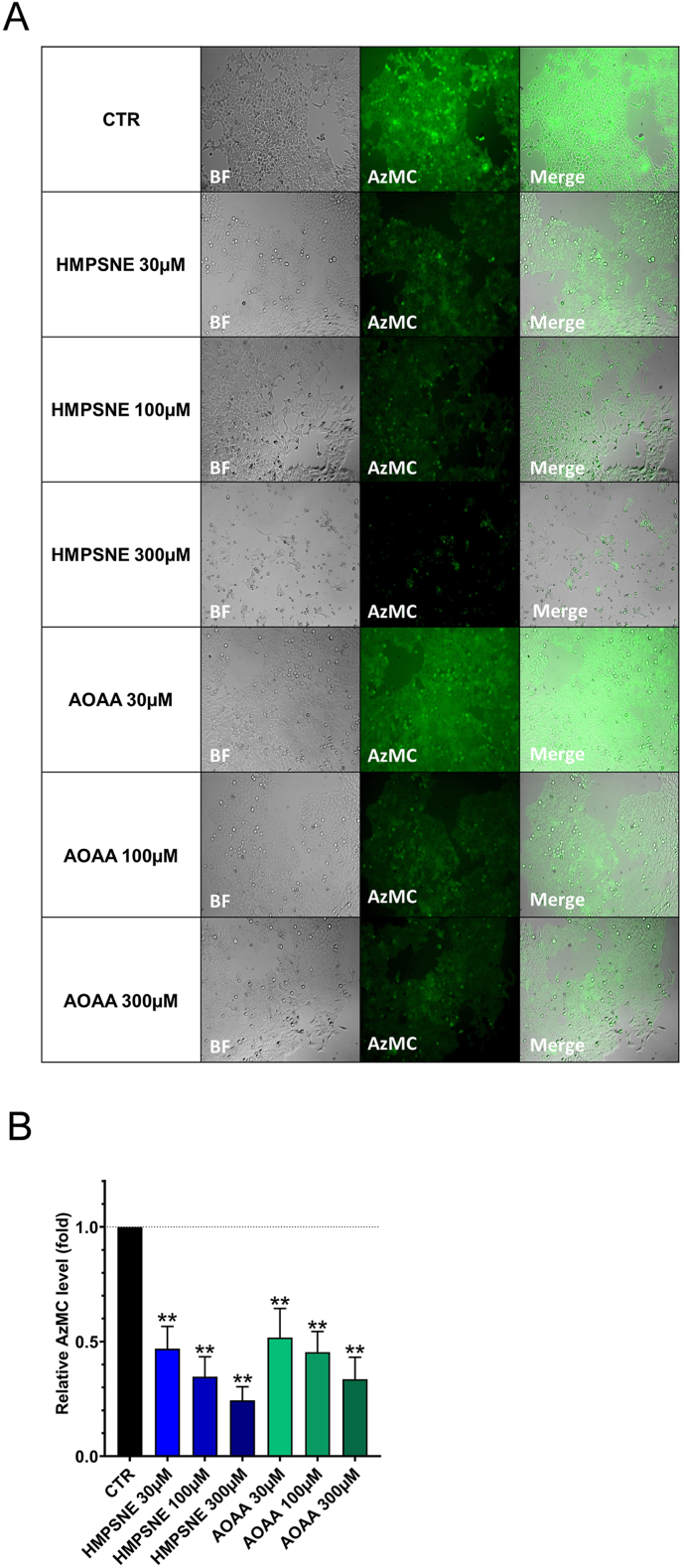
**H**_**2**_**S quantification in live HCT116 cells in presence of HMPSNE or AOAA.** The remaining H_2_S in the cell after 48 h incubation with several concentrations of HMPSNE or AOAA was quantified by fluorescence imaging. **A:** Representative pictures showing brightfield (BF), AzMC and merged images. **B**: Quantification of AzMC fluorescence per cell. Data are shown as mean ± SEM, n = 3, **p<0.01 compared to control.

**Fig. 3:**
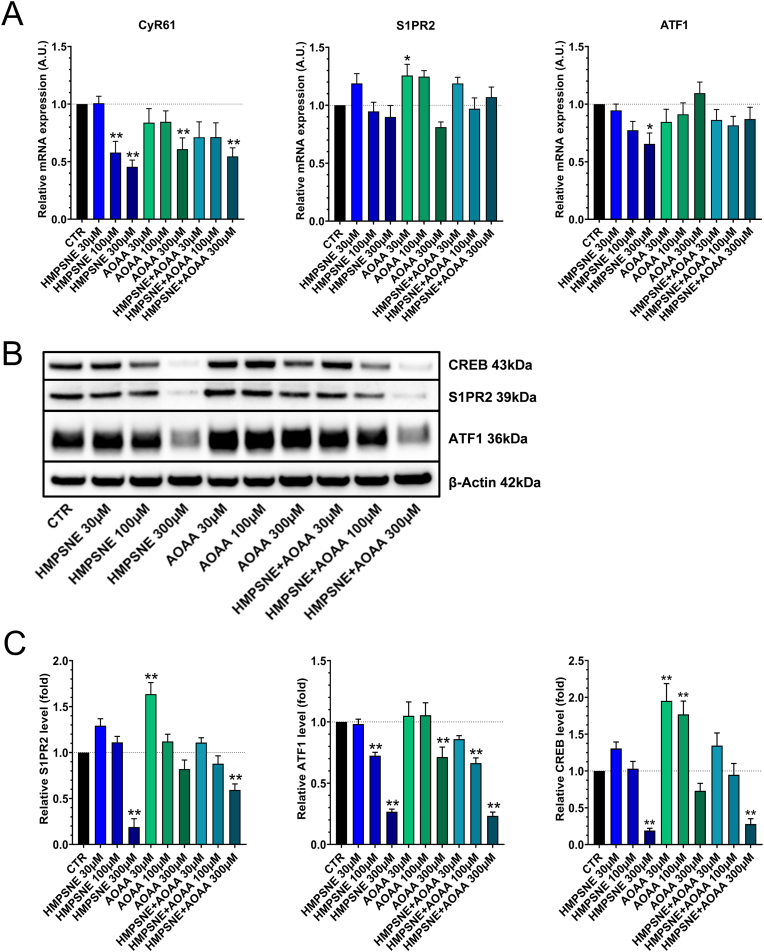
**3-MST inhibition suppresses CyR61 mRNA levels through the inhibition of S1PR via ATF1 and CREB (p38MAPK pathway). A**: Quantitative real-time PCR (qPCR) analysis of endogenous mRNA levels of CyR61, S1PR2 and ATF1 in HCT116 cells after incubation with HMPSNE, AOAA or their combination for 48 h. Data are shown as mean ± SEM of at least 4 independent experiments, *p < 0.05, **p < 0.01 compared to control. **B**,**C**: Western blot analysis of S1PR2 and p38MAPK pathway markers in presence of HMPSNE, AOAA or their combination for 48 h. Data are shown as mean ± SEM, n = 4, **p<0.01 compared to control.

**Inhibition of 3-MST suppresses CyR61 mRNA expression through S1PR via ATF1 and CREB**. Since our data indicated that H_2_S and polysulfides are able to induce the activation of the CyR61 promoter, we next examined the effect of H_2_S biosynthesis inhibition on CyR61 mRNA expression in HCT116 cells. Cells were treated with various concentrations of prototypical H_2_S biosynthesis inhibitors for 48 h. We used HMPSNE for inhibition of 3-MST; and AOAA for inhibition of CBS. We have also tested the combined application of both agents – since in some cases after inhibition of one biological source of H_2_S, cellular responses can be partially compensated by another H_2_S producing enzymes [1,15]. Using live cell imaging, we quantified H_2_S production in HCT116 cells after treatment of the cells with HMSPNE or AOAA. There was a significant concentration-dependent decrease of H_2_S levels in response to both inhibitors (Fig. 2). We have also quantified CyR61 mRNA levels after treatment of the cells with HMSPNE or AOAA and found that each inhibitor, as well as the combination of these two inhibitors significantly decreased CyR61 mRNA levels (Fig. 3A).

Next, we have conducted a series of experiments to investigate the mechanism involved in the above effect. Previous studies have demonstrated that CyR61 expression can be upregulated via S1PR (sphingosine 1-phosphate receptor), RhoA GTPase and p38MAPK signaling [45,48]. Thus, we have investigated the expression of S1PR (sphingosine 1-phosphate receptor), CREB and ATF1 (p38MAPK signaling pathway) in the current experimental system and tested the effect of H_2_S biosynthesis inhibition. Although HMPSNE had no significant effect on S1PR2 mRNA, a significant downregulation of S1PR2 protein level was observed after treatment of the cells with the 3-MST inhibitor. In contrast, the CBS inhibitor AOAA significantly increased S1PR2 mRNA and protein levels. When the two inhibitors were tested in combination, a significant decrease of S1PR2 protein level was noted (Fig. 3A,B,C). ATF1 protein was significantly downregulated in response to either of the two inhibitors as well as in response to their combination, although the inhibitory effect of these agents on its mRNA was relatively slight (Fig. 3A,B,C). Similarly to S1PR2, CREB protein was also downregulated by treatment of the cells with the 3-MST inhibitor HMPSNE, while AOAA did not exert an inhibitory effect at high concentrations, and it exerted stimulatory effects at lower concentrations (Fig. 3B and C).

**Fig. 4:**
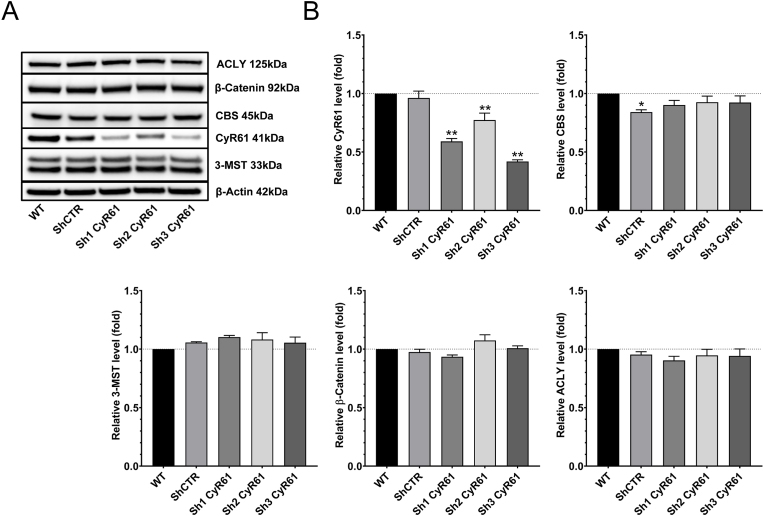
**CyR61 does not regulate the expression of H**_**2**_**S-producing enzymes or the Wnt/β-catenin pathway. A**,**B**: Western blot analysis of CyR61, H_2_S enzymes and Wnt/β-Catenin pathway markers in wild-type HCT116 cells, ShCTR cells (i.e. cells treated with non-coding vector) and three HCT116 cell lines with CyR61 silencing. Data are presented as mean ± SEM of at least 4 independent experiments. *p<0.05, **p<0.01 compared to control.

**CyR61 does not regulate CBS or 3-MST expression, nor does it regulate the Wnt/β-catenin pathway.** To assess the potential role of CyR61 in the regulation of H_2_S-producing enzymes, we have tested the effect of CyR61 knockdown on the expression of CBS and 3-MST. Introduction of a viral vector containing shRNAs targeting the CyR61 gene into HCT116 cells yielded a 60% downregulation of CyR61 protein. However, CyR61 silencing did not affect the expression of either CBS or 3-MST. β-catenin expression, or the expression of ACLY protein (which is involved in the regulation of the Wnt/β-catenin pathway) were also unaffected by CyR61 silencing (Fig. 4A and B).

**Fig. 5:**
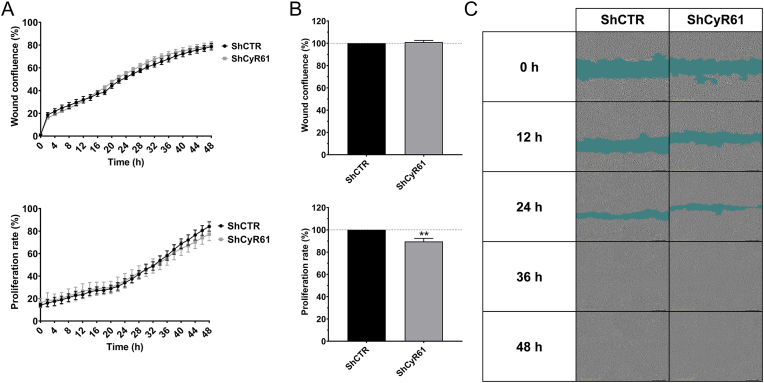
**CyR61 silencing slightly suppresses HCT116 cell proliferation and has no effect on cell migration. A**: Representative curves of wound confluence and proliferation rates in percentage of HCT116 ShCTR and HCT116 ShCyR61 cell lines. **B**: Quantitative analysis of wound healing rates and proliferation rates for 48 h. Data are shown as mean ± SEM, n = 5, **p<0.01 compared to control. **C**: Migration of HCT116 ShCTR and HCT116 ShCyR61 cells, assessed by wound healing assays at 48 h.

**CyR61 silencing attenuates colon cancer cell proliferation, but does not affect migration**. Next, we have examined if the CyR61 silencing modulates HCT116 migration or proliferation. We have selected the HCT116 Sh3 CyR61 cell line with the strongest CyR61 downregulation and performed an IncuCyte Scratch Wound Assay and an IncuCyte Cell Count Proliferation Assay. CyR61 silencing produced a slight, but statistically significant suppression of cell proliferation. However, it did not affect cell migration (Fig. 5A,B,C).

**Fig. 6:**
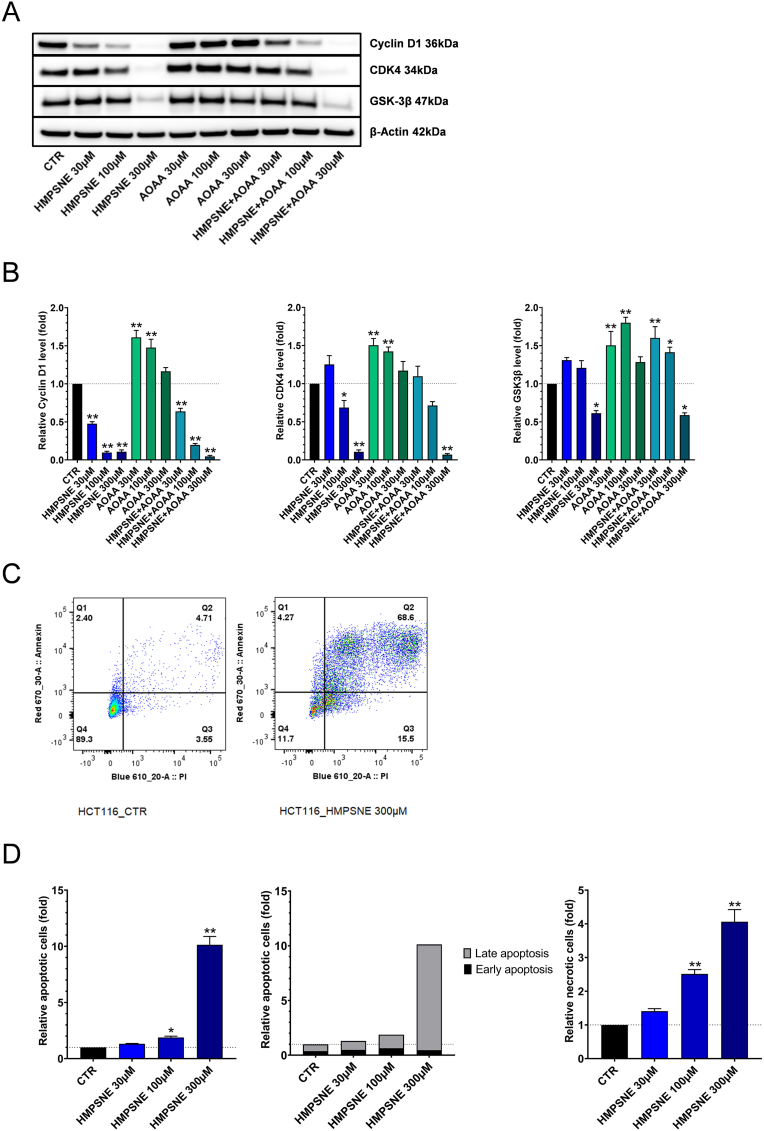
**3-MST inhibition in HCT116 cells induces cyclin D1 downregulation in part via ATF1, CREB and CDK4, which is associated with G1-S phase cell arrest and cell death. A**,**B**: Western blot analysis of Cyclin D1, CDK4 and GSK3β proteins after treatment of HCT116 cells with HMPSNE, AOAA or their combination for 48 h. Data are shown as mean ± SEM, n = 4, **p < 0.01 compared to control. **C**: Representative FACS plots showing the Q1 population (early apoptotic cells), Q2 population (late apoptotic cells), Q3 population (necrotic cells) and Q4 population (cells alive). **D**: Quantification of apoptotic cells, distinguishing the late and the earlier apoptosis ratios, and necrotic cells compared to control. Data are shown as mean ± SEM, n = 5, *p<0.05, **p<0.01 compared to control.

**Cyclin D1 downregulation in part via ATF1, CREB and CDK4 promotes cell cycle arrest in G1-S phase, leading to apoptosis and necrosis.** The transcription factors AP-1, composed by ATF, JUN and FOS, and CREB can activate the Cyclin D1 promoter, a key player in G1-S phase transition [47,[55], [56], [57]]. Since 3-MST inhibition suppresses ATF1 and CREB protein expression (Fig. 3A,B,C), we have examined if HMPSNE also affects cyclin D1 expression. HMPSNE – alone or combined with AOAA – caused a significant downregulation of cyclin D1, when both agents were used at their highest concentrations (Fig. 6A and B). This downregulation can be explained in part by a decrease in ATF1 and CREB expression. However, it does not explain why Cyclin D1 is downregulated at lower HMPSNE concentrations, where the expression of ATF1 or CREB is not decreased. This finding indicates that others signalling processes may also contribute to the regulation of Cyclin D1 in the current experimental system. Thus, we next measured the expression of two other molecules, CDK4 and GSK-3β – both of them being known regulators of Cyclin D1 activation [[55], [56], [57], [58]]. Indeed, at high concentrations of HMPSNE, CDK4 was found to be downregulated and therefore may be potentially involved in the regulation of Cyclin D1.

When Cyclin D1 is downregulated, cell cycle arrest is known to occur [[59], [60], [61]], which, in turn, can induce apoptotic cell death [62]. To examine if HMPSNE promotes apoptosis and/or necrosis in HCT116 cells, we used the APC Annexin V Apoptosis Detection Kit with Propidium Iodide Solution. This assay can discriminate cells in early apoptosis (annexin-positive and PI-negative cells), late apoptosis (annexin- and PI-positive cells) and necrosis (annexin-negative and PI-positive cells). Our results show that HMPSNE, at the higher concentrations used, leads to a significant increase in the apoptotic cell population, mainly representing the late form of apoptosis; it also increases the necrotic cell population (Fig. 6C and D). It is important to notice that, at lower HMPSNE concentration (30 μM) despite a significant downregulation of Cyclin D1, the activation of apoptosis was not statistically significant – although a trend for an increase was already noted. At this concentration of the inhibitor, CREB, CDK4 and GSK-3 were found to be upregulated. Thus, when 3-MST activity is only partially inhibited, the effects on various signalling pathways may be variable; the effects may be functionally diverse, which, ultimately, only exerts a slight net effect on the process of apoptosis.

**Fig. 7:**
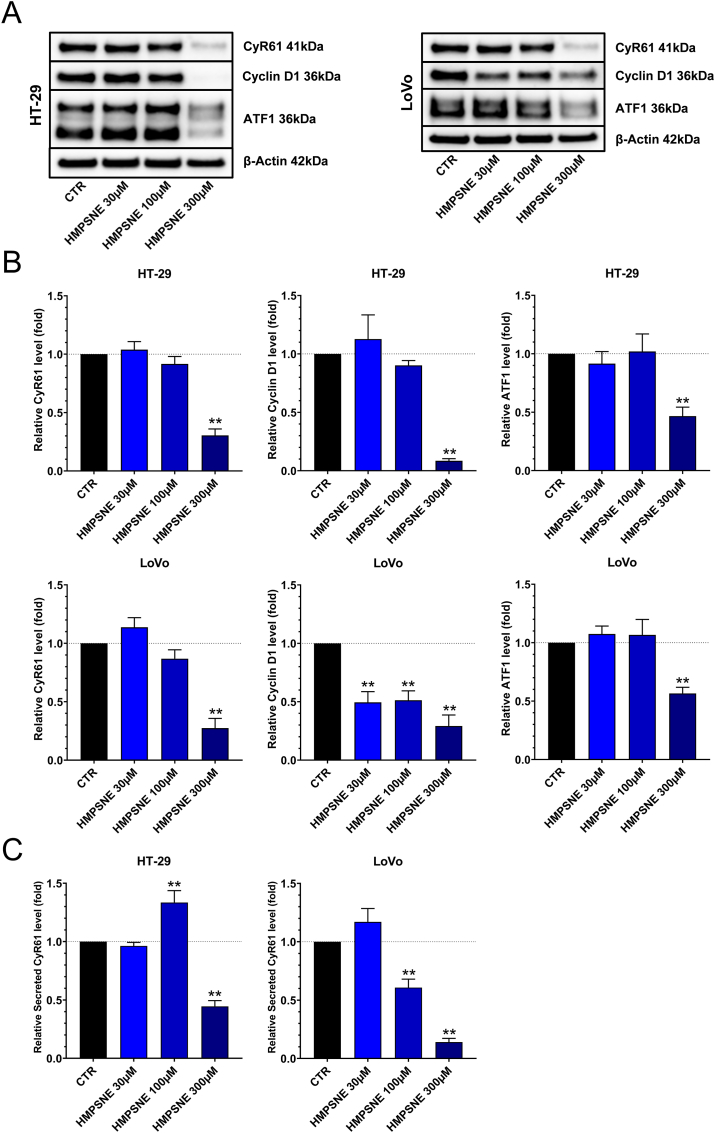
**3-MST inhibition downregulates CyR61 protein levels and promotes cell cycle arrest in HT-29 and LoVo cells. A**,**B:** Western blot analysis of CyR61, Cyclin D1 and ATF1 proteins after incubation with HMPSNE for 48 h. Data are shown as mean ± SEM, n = 3, **p < 0.01 compared to control. **C:** ELISA of secreted CyR61 after incubation with HMPSNE for 48 h. The absolute concentration of CyR61 in the control supernatant (in the absence of pharmacological inhibitors was 54 ± 9 and 822 ± 234 pg/ml in HT-29 and LoVo cells, respectively. Data are shown as mean ± SEM, n = 4, **p<0.01 compared to control.

**Fig. 8:**
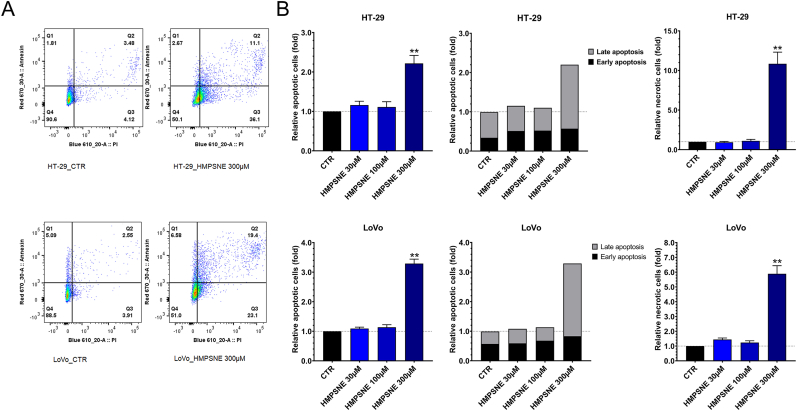
**3-MST inhibition promotes apoptosis and necrosis in HT-29 and LoVo cells**. **A**: Representative FACS plots showing the Q1 population (early apoptotic cells), Q2 population (late apoptotic cells), Q3 population (necrotic cells) and Q4 population (cells alive). **B**: Quantification of apoptotic cells, distinguishing the late and the earlier apoptosis ratios, and necrotic cells, compared to control. Data are shown as mean ± SEM, n = 5, **p<0.01 compared to control.

In order to extend our findings to other colon cancer cells, we have also tested the effect of the 3-MST inhibitor on HT-29 and LoVo cells, two additional human colon cancer cell lines which are known to produce H_2_S from CBS and 3-MST. HMPNSE caused the downregulation of cyclin D1, most likely via downregulation of ATF1 in these cells (Fig. 7A and B) and increased the apoptotic and necrotic cell populations (Fig. 8A and B). In HCT116 cells, HMPSNE tended to induce more apoptosis than necrosis, while in LoVo and HT-29 cells it induced more necrosis and less apoptosis (Fig. 6C and D and Fig. 8A and B).

**Fig. 9:**
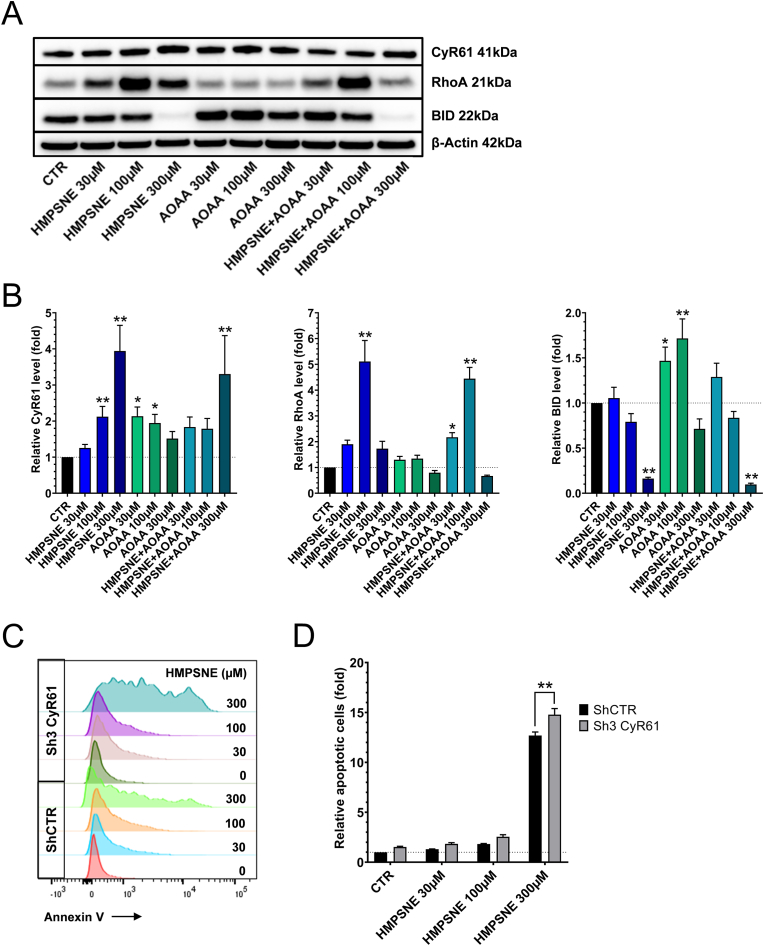
**3-MST inhibition induces BID downregulation and CyR61 upregulation in part via RhoA. Endogenously produced CyR61 acts as a negative regulator of apoptosis in HCT116 cells treated with 3-MST inhibitor**. **A**,**B**: Western blot analysis of BID, CyR61 and RhoA proteins in HCT116 cells treated with HMPSNE, AOAA or their combination for 48 h. Data are presented as mean ± SEM of at least 5 independent experiments. *p < 0.05, **p < 0.01 compared to control. **C**: Histogram showing the annexin-positive cell population in HCT116 ShCTR and Sh3 CyR61 after 48 h incubation with HMPSNE. **D**: Comparison of the apoptotic cell population in HCT116 ShCTR vs. Sh3 CyR61 after treatment of the cells with HMPSNE for 48 h. Data are shown as mean ± SEM, n = 5, **p<0.01 compared to control.

**Fig. 10:**
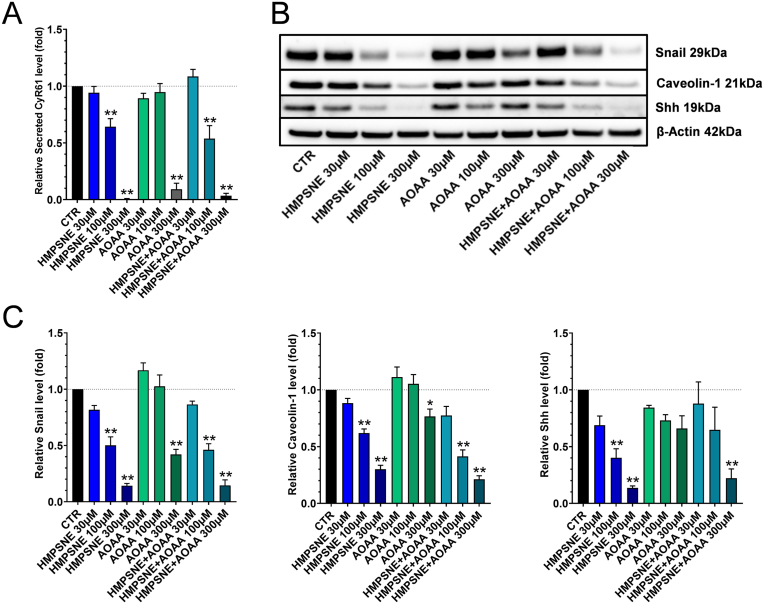
**3-MST inhibition results in Shh downregulation and inhibits CyR61 secretion via the suppression of Snail and Caveolin-1 expression. A**: ELISA of CyR61 levels in the cell culture supernatant after incubation of HCT116 cells with HMPSNE, AOAA or their combination for 48 h. Data are shown as mean ± SEM, n = 4, **p < 0.01 compared to control. The absolute concentration of CyR61 in the control supernatant (in the absence of pharmacological inhibitors was 275 ± 24 pg/ml. **B**,**C**: Western blot analysis of Caveolin-1, Snail and Shh proteins after treatment of HCT116 cells with HMPSNE, AOAA or their combination for 48 h. Data are shown as mean ± SEM, n = 4, *p<0.05, **p<0.01 compared to control.

**3-MST inhibition induces BID downregulation and CyR61 upregulation in part via RhoA, thereby modulating apoptosis**. Since we have observed that HMPSNE or AOAA decrease CyR61 mRNA expression (Fig. 3A), we expected that a consequent downregulation of the CyR61 protein would also occur. However, HMPSNE and AOAA significantly *increased* CyR61 proteins levels (Fig. 9A and B). We have, in turn, investigated the mechanism underlying this unexpected response. Our results show that HMPSNE (but not AOAA) increased RhoA protein level (Fig. 9A and B). Since this protein is known to be involved in the stimulation of CyR61 expression [45,63], we hypothesize that RhoA expression may be an additional effect of 3-MST inhibition, which, in turn, counterbalances the suppression of CyR61 mRNA levels. The reduction of CyR61 mRNA is more pronounced with HMPSNE than with AOAA; this difference may explain why RhoA expression in response to AOAA is upregulated to a smaller degree than in response to HMPSNE (Fig. 3A). It is important to mention that RhoA can only partially explain the upregulation of CyR61. At lower concentrations of HMPSNE (100 μM) RhoA appears to be the principal player in CyR61 upregulation, but its contribution to the decrease in CyR61 secretion appears to be slighter. However, at higher HMPSNE concentrations, the slight upregulation of RhoA cannot explain the marked upregulation of CyR61: in this case we observed a marked blockade of CyR61 secretion, which, in turn, is reflected in a marked accumulation of CyR61 inside the cell (Fig. 10).

Next, we have tested the hypothesis that the upregulation of CyR61 in colon cancer cells serves to prevent apoptosis. When HCT116 ShCTR and HCT116 Sh3 CyR61 cells were treated with HMPSNE for 48 h, the number of apoptotic cells was found to be higher in the CyR61 silencing cell line than in the wild-type control cells, suggesting that CyR61 may serve to prevent or delay apoptosis (Fig. 9C and D). RhoA upregulation and BID downregulation were also observed after HMPSNE treatment (Fig. 9A and B); we hypothesize that these effects may also participate in the development of apoptosis resistance, consistent with such function of these proteins in other cell types [64,65]. Contrary to HCT116 cells, 300 μM of HMPSNE significantly *decreased* CyR61 proteins levels in HT-29 and LoVo cells (Fig. 7A and B), highlighting a colon cancer cell-type-dependence of the regulation of CyR61 by the 3-MST system.

**Pharmacological inhibition of H**_**2**_**S biosynthesis suppresses Snail and Caveolin-1 expression, downregulates Shh and inhibits CyR61 secretion.** CyR61 is present in two forms: cell-associated as well as a secreted/circulating form, with the latter being considered a soluble, circulating mediator, as well as a biomarker [[26], [27], [28], [29], [30]]. In order to investigate if pharmacological inhibition of 3-MST or CBS affects the secretion/release of CyR61 from HCT116 cells, we incubated HCT116 cells with various concentrations of HMPSNE or AOAA (either alone, or in combination) for 48 h and then performed an ELISA to quantify soluble CyR61 levels in the cell culture supernatant. HMPSNE and AOAA both exerted a marked suppressive effect on the secretion of CyR61 in HCT116 cells (Fig. 10A). Similar effect was also observed in HT-29 and LoVo cells (Fig. 7C).

To assess which molecules could be involved in the HMPSNE- or AOAA-induced inhibition of Cyr61 release, we have measured Snail and Caveolin-1 expression, two molecules that are known to be involved in the promotion of CyR61 secretion from various cancer cells [28,66]. Fig. 10B and C shows that both of these proteins are downregulated after treatment of the cells with HMPSNE and to a smaller extent after treatment of the cells with AOAA. HMPSNE also resulted in the inhibition of the expression of Sonic Hedgehog (Shh) protein (Fig. 10B and C). This protein – an endogenous ligand of the hedgehog pathway – has also been implicated previously in the regulation of CyR61 in various forms of cancer [67,68].

## 3 Discussion

In separate sets of studies, the significant pathophysiological role of **(a)** the upregulation of various H_2_S-producing enzymes and **(b)** CyR61 biosynthesis and secretion has been already defined in various forms of cancer, including colon cancer (see Introduction). However, the potential relationship between H_2_S and the CyR61 pathway has not yet been examined, and the current project was designed to examine a potential connection between them. The fact that in colorectal cancer, on one hand, H_2_S and polysulfides are overproduced, and on another hand, CyR61 is overexpressed, led us to speculate that increased H_2_S levels, due to the induction of various H_2_S-producing enzymes in cancer cells, may induce or activate CyR61, which, in turn, may contribute to cancer aggressiveness. The results of the current report show that H_2_S and polysulfides can, indeed, activate the CyR61 promoter (Fig. 1A,B,C), and HMPSNE and AOAA suppress CyR61 mRNA levels (Fig. 3A) in various colon cancer cell types. However, the data also demonstrated that this activation does not always or necessarily yield higher CyR61 intracellular protein levels, due to a combination of reasons (see below).

What, then, is the molecular mechanism of CyR61 promoter activation by 3-MST? We do not currently know if this activation reflects H_2_S/polysulfide overproduction, followed by a direct biding of these reactive species to promoter elements, or if this effect is due to a possible activation or modification of regulators that bind to the CyR61 promoter. An important global regulatory mechanism triggered by H_2_S/polysulfides is S-sulfhydration (also termed persulfidation), a fundamental posttranslational mechanism which can affect hundreds or thousands of proteins in various mammalian cells [1,[69], [70], [71], [72]]. Although S-sulfhydration was originally linked to H_2_S, it is now well accepted that this modification is principally mediated by polysulfide chemistry (and not by H_2_S or HS^−^). When a H_2_S donor (like the slow-acting donor GYY4137 in our experiments) is applied to a cell, nevertheless, it will generate a mixture of polysulfides and H_2_S [73,74]. The fact that in the current experiments both H_2_S and the polysulfide donor (Na_3_S_2_) induced concentration-dependent inductions of the CyR61 promoter suggests that a sulfhydration mechanism may be involved. Indeed, prior studies have demonstrated that various promoters can be regulated by sulfhydration; for instance the Nrf2/ARE pathway is regulated by sulfhydration of Keap1 [75]. Various elements of the NF-κB pathway (IKKβ, as well NF-κB itself) have also been shown to be subject to sulfhydration [76,77]. Moreover, Sp1 – a known activator of the CyR61 promoter – was also found to be subject of S-sulfhydration, which, in turn, was shown to regulate mRNA expression [[78], [79], [80]]. Thus, our current working hypothesis is that endogenously generated H_2_S (most likely via intermediary polysulfides) induces CyR61 mRNA through Sp1 sulfhydration in colon cancer cells.

In order to determine if CyR61 levels are, indeed, regulated by *endogenously produced* H_2_S, we have applied AOAA and HMPSNE. These compounds are standard pharmacological inhibitors of the principal H_2_S-producing enzymes CBS and 3-MST, respectively. We have selected appropriate concentrations of these agents, where these compounds produce initially a partial, and then a complete inhibition of H_2_S synthesis from their respective enzymatic sources [20]. Both inhibitors produced a significant inhibition of CyR61 mRNA levels (Fig. 3A), indicating the importance of endogenous H_2_S/polysulfides in the upregulation of the CyR61 promoter in colon cancer cells.

Since the CyR61 promoter has been shown to be activated by S1P through RhoA GTPase and p38MAPK signaling pathways [[45], [46], [47], [48], [49]], we have also quantified the effect of H_2_S biosynthesis inhibition on the expression of S1PR, ATF1 and CREB. Our results show that these molecules are also downregulated by HMPSNE (Fig. 3A,B,C). Thus, we conclude that endogenous H_2_S/polysulfide biosynthesis by 3-MST in colon cancer cells promotes CyR61 mRNA induction, most likely through Sp1 sulfhydration, and through the activation of S1PR, ATF1 and CREB. The finding that the 3-MST inhibitor exerted more pronounced effects than the CBS inhibitor is consistent with the fact that 3-MST is the more significant biological source of polysulfides while CBS predominantly generates “free” H_2_S [1,81].

While the H_2_S system regulates the activation of the CyR61 promoter, CyR61 does not appear to regulate the expression of the two principal H_2_S-producing enzymes in HCT116 cells (Fig. 4A and B). Previous studies have demonstrated that CyR61 upregulation can activate the Wnt/β-catenin pathway in gliomas [52] and that β-catenin activation or overexpression can upregulate CyR61 in hepatocellular carcinomas and pancreatic cancers [53,54]. However, the influence of CyR61 in Wnt/β-catenin activation in colon cancer has not been examined previously. Our results show that CyR61 does not promote the activation of the Wnt/β-catenin pathway activation in colon cancer cells (Fig. 4A and B). However, the partial nature of the CyR61 silencing achieved (60% downregulation) must be taken into consideration when interpreting these results; it is possible that a more complete downregulation or complete deletion of CyR61 may have produced a different effect.

CyR61 overexpression in colon cancer cells leads to increased cell migration, invasion and high CyR61 in cancer patients and is associated with poor clinical prognosis [26,[40], [41], [42], [43], [44]]. In our experiments, partial CyR61 silencing had no significant impact on HCT116 migration and only exerted a very slight inhibitory effect on cell proliferation (Fig. 5A,B,C). Once again it is possible that the remaining 40% of CyR61 after CyR61 silencing may be sufficient to maintain the functionality of the CyR61 system in regulating proliferation and migration. Alternatively, there may be a compensatory upregulation of other effector pathways that may compensate after CyR61 silencing and maintain functionality of these cells. Further studies, e.g. using a cell line with a complete deletion of this protein, would be needed to further explore the role of CyR61 in the regulation of HCT116 proliferation and migration.

The HMPSNE-induced downregulation of ATF1 and CREB (Fig. 3A,B,C) coincided with a decrease in Cyclin D1 protein expression (Fig. 6A and B). We hypothesized that this event may promote a cell cycle arrest in G1/S phase. Masamha and Benbrook have previously observed that Cyclin D1 degradation is sufficient to induce G1 cell cycle in ovarian cancer cells [58]. Radu and colleagues have reported that the downregulation of Cyclin D1 induced by PTEN promotes cell G1 cycle arrest in glioblastomas [59]. In addition, O’Connor et al. demonstrated that poly(ADP-ribose) polymerase family member 14 downregulation decreases Cyclin D1 and promotes G1 cell cycle arrest [60]. Since prolonged cell cycle arrest can culminate in cell death [61] we have investigated the effect of 3-MST inhibition on the apoptotic/necrotic state of the HCT116 cells (as well as a number of additional human colon cancer cell lines). Our results confirm that the Cyclin D1 downregulation increases late apoptosis and necrosis, with the relative extent of the two forms of cell death being cell-type dependent (Fig. 6C, **7A,B,8A,B**). Moreover, our data (Fig. 9C and D) suggest that CyR61 may play a role in preventing or retarding the apoptosis in response to 3-MST inhibition. This conclusion is based on the finding that CyR61 silencing in HCT116 cells increases the portion of apoptotic cells in response to HMPSNE incubation.

Importantly, 3-MST inhibition decreased CyR61 mRNA levels, but did not always suppress cell-associated CyR61 protein levels; in HCT116, in fact, a paradoxical *increase* was noted (Fig. 9A and B) – while in HT-29 and LoVo cells a decrease was observed (Fig. 7A and B). Upregulation of cell-associated CyR61 in HCT116 cells may be explained on one hand by the increased expression of RhoA proteins (Fig. 9A and B), known to be involved in the stimulation of CyR61 expression [45,62], which may protect the CyR61 mRNA and the protein from degradation, i.e. prolongs their half-life. On the other hand, the increase of cell-associated CyR61 protein levels after HMPSNE treatment in HCT116 cells may also be related to the fact that 3-MST inhibition blocks the secretion/release of CyR61 into the cell culture supernatant (Fig. 10A), thereby retaining more protein in the cell-associated component. We speculate that such an effect may be therapeutically beneficial, since circulating CyR61 protein may exert pro-angiogenic, pro-inflammatory and immune-cell-modulatory effects [[82], [83], [84], [85], [86]] that are likely tumor-supportive in their nature.

As already mentioned above, the current study demonstrates that the H_2_S/CyR61 interactions are not only (likely) different in different forms of cancer, but *even are heterogeneous when comparing different human colon cancer cell lines.* Contrary to HCT116 cells, HMPSNE significantly reduced cell-associated CyR61 protein levels in HT-29 and LoVo cells (Fig. 7A and B). This difference may explain the different ratios apoptotic/necrotic cells observed between HCT116 versus HT-29 and LoVo cells, HMPSNE promoting mainly apoptosis in HCT116 cells and mainly inducing necrosis in HT-29 and LoVo cells. Besides CyR61, RhoA upregulation and BID downregulation after 3-MST inhibition (Fig. 9A and B), may also contribute to the development of apoptosis resistance. Indeed, in prior studies Zhang and Jiang described that RhoA inhibits the hypoxia-induced apoptosis in chondrocytes [63] and Khaider and colleagues demonstrated that the inhibition of BID expression by Akt leads to resistance to TRAIL-induced apoptosis in ovarian cancer cells [64]. Our findings, demonstrating that CyR61 in HCT116 cells plays an important role in cell survival after induction of apoptosis, are conceptually in line with prior studies concluding that CyR61 confers cytoprotective effects – including chemotherapy resistance – in various forms of advanced cancers including colorectal [[87], [88], [89], [90]].

The inhibitory effect of H_2_S biosynthesis inhibition on CyR61 secretion into the supernatant was very prominent, and this response was consistent across all human colon cancer cell lines studied (Fig. 7, Fig. 10A). Snail and Caveolin-1 are known to be involved in CyR61 secretion [28,65]; both of these proteins were found to be downregulated with HMPSNE or by, AOAA (Fig. 10B and C). The suggestion that Snail downregulation is involved in the suppression of Cyr61 secretion is consistent with the results of Tanaka and colleagues who demonstrated that Snail promotes CyR61 secretion in squamous cell carcinoma [28]. In our study, we have also detected a decrease of Shh protein after inhibition of 3-MST in HCT116 cells (Fig. 10B and C). This protein, as a ligand of the sonic hedgehog pathway – similar to CyR61 and Caveolin-1 – is also involved in the stimulation of cancer cell proliferation and migration [[91], [92], [93]]. Downregulation of these molecules may contribute to the previously reported [20] suppression of HCT116 cells migration after pharmacological inhibition of H_2_S biosynthesis.

## 4 Conclusions and implications

**Fig. 11:**
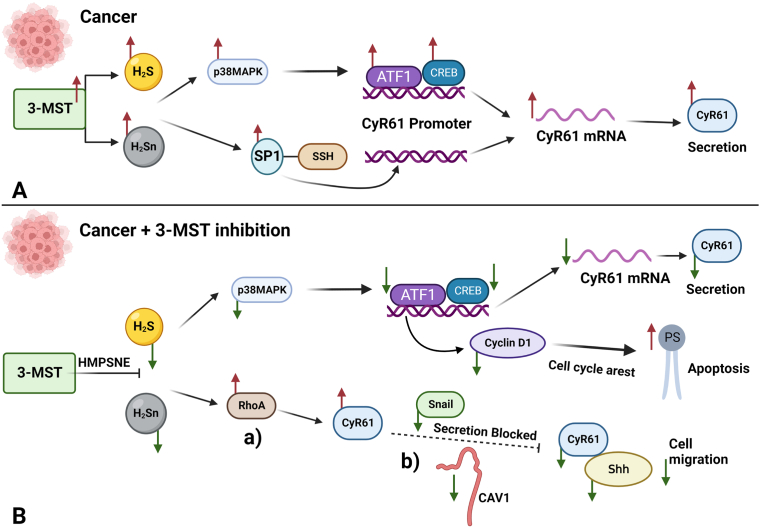
**Modulation of the CyR61 pathway by endogenously produced 3-MST related H**_**2**_**S/polysulfides.** Modulation of the CyR61 pathway by endogenously produced H_2_S/polysulfides: a working hypothesis. **A**: Endogenous, 3-MST-derived H_2_S and H_2_Sn biosynthesis in cancer cells induces Sp1 sulfhydration, S1PR activation and induces the p38MAPK signaling pathway through ATF1 and CREB activation, leading to increased levels of CyR61 mRNA and CyR61 protein. **B**: The reduction of H_2_S and H_2_Sn biosynthesis, achieved by a pharmacological 3-MST inhibitor, suppresses this pathway. The reduction of ATF1 mRNA and protein levels after inhibition of 3-MST inhibition leads to Cyclin D1 downregulation, promoting cell cycle arrest and inducing apoptosis. Although inhibition of H_2_S and H_2_Sn biosynthesis suppresses CyR61 mRNA, this effect is not always reflected in a decrease in cell-associated CyR61 protein levels, which show a heterogeneous response (an increase in HCT116 cells and a decrease in HT-29 and LoVo cells). This is likely due to the combination of two effects: (a) after 3-MST inhibition, there is an increase of RhoA proteins in the cells, which can stabilize CyR61 mRNA and protein and (b) 3-MST inhibition produces a blockade of CyR61 secretion, thereby retaining some of this protein in the cell-associated fraction. The latter effect is likely related to the suppression of Snail and Caveolin-1, which have a known role in the stimulation of CyR61 secretion in cancer cells. The decrease of Caveolin-1, CyR61 secretion and Shh, contribute to the decreased HCT116 migration demonstrated in our previous study [20]. Finally, when CyR61 is retained in the cell, it plays a role as an anti-apoptotic factor.

The main conclusions of the current study can be integrated into the working hypothesis outlined in Fig. 11. According to this hypothesis, **(1)** endogenous, 3-MST-derived H_2_S and H_2_Sn biosynthesis in cancer cells induces Sp1 sulfhydration, S1PR activation and induces the p38MAPK signaling pathway through ATF1 and CREB activation. **(2)** The reduction of H_2_S and H_2_Sn biosynthesis, in response to pharmacological inhibition of 3-MST, suppresses this pathway. **(3)** The reduction of ATF1 mRNA and protein levels after inhibition of H_2_S and H_2_Sn biosynthesis through 3-MST inhibition leads to cyclin D1 downregulation, promoting cell cycle arrest and inducing apoptosis. **(4)** Although inhibition of H_2_S and H_2_Sn biosynthesis through 3-MST inhibition suppresses CyR61 mRNA, this effect is not always reflected in a decrease in cell-associated CyR61 protein levels, which show a heterogeneous response (an increase in HCT116 cells and a decrease in HT-29 and LoVo cells). This is likely due to the combination of two effects: **(5)** After 3-MST inhibition, there is an increase of RhoA proteins in the cells, which can stabilize CyR61 mRNA and protein and **(6)** 3-MST inhibition produces a blockade of CyR61 secretion, thereby retaining some of this protein in the cell-associated fraction. **(7)** The latter effect is likely related to the suppression of Snail, Caveolin-1 and Shh proteins, which all have a known role in the stimulation of CyR61 secretion in cancer cells. Finally, **(8)** when CyR61 is retained in the cell, it plays a role as an anti-apoptotic factor.

In conclusion, the current study, by unveiling the regulation of CyR61, tumor-derived H_2_S and H_2_Sn produced by 3-MST, identified an additional pathway through which cancer cells utilize H_2_S and H_2_Sn to their benefit. This mechanism adds to the list of mechanisms through which pharmacological inhibition of cancer cell H_2_S and H_2_Sn biosynthesis may exert antitumor effects.

## 5 Methods

**Cell Culture.** The HCT116 human colorectal carcinoma was obtained from ATCC (American Type Culture Collection, Manassas, VA, USA), HT-29 and LoVo human colorectal adenocarcinoma cell lines were purchased from DSMZ (German Collection of Microorganisms and Cell Cultures GmbH, Braunschweig, Germany). HCT116 and HT-29 cells were cultured in McCoy's 5A (Modified) Medium (Gibco, Thermo Fisher Scientific, Waltham, MA, USA) supplemented with 10% FBS (Gibco, Thermo Fisher Scientific), 100 units/ml of penicillin and 100 μg/ml of streptomycin (Gibco, Thermo Fisher Scientific). LoVo cells were cultured in Ham's F–12K Medium (Gibco, Thermo Fisher Scientific) with 10% FBS (Gibco, Thermo Fisher Scientific), 100 units/ml of penicillin and 100 μg/ml of streptomycin (Gibco, Thermo Fisher Scientific).

**Reagents and antibodies.** As previously described in Ref. [20], we used the 3-MST inhibitor HMPSNE (2-[(4-hydroxy-6-methylpyrimidin-2-yl)sulfanyl]-1-(naphthalen-1-yl)ethan-1-one) the CBS inhibitor AOAA (O-(carboxymethyl)hydroxylamine hemihydrochloride) and the H_2_S donor GYY4137 [4-methoxyphenyl(morpholino)phosphinodithioate morpholinium salt]. The sodium trisulfide (Na_2_S_3_) was obtained from Dojindo Molecular Technologies, Inc. (Rockville, MD, USA).

Rabbit monoclonal anti-CBS (D8F2P), anti-ACLY (D1X6P), anti-β-catenin (D10A8), anti-Cyclin D1 (E3P5S), anti-CYR61 (D4H5D), anti-RhoA (67B9), Snail (C15D3), Caveolin-1 (D46G3), Shh (C9C5), CREB (48H2), rabbit polyclonal anti-BID antibody (Human Specific) and anti-mouse IgG HRP-linked antibody were purchased from Cell Signaling Technology (Danvers, MA, USA). Mouse monoclonal anti-β-actin (AC-15) was obtained from Sigma-Aldrich (Burlington, MA, USA). Anti-rabbit IgG (H + L) cross-adsorbed secondary antibody-HRP was purchased from Invitrogen (Thermo Fisher Scientific). Rabbit polyclonal anti-3-MST (ab224043), anti-S1PR2 (ab235919) and rabbit monoclonal anti-ATF1 (ab134104) were acquired from Abcam (Cambridge, England).

**H**_**2**_**S detection using AzMC-aided live cell imaging**. The H_2_S sensitive fluorescent probe 7-azido-4-methylcoumarin (AzMC) was used to quantify H_2_S levels in the cells, as previously described [94]. HCT116 cells were seeded in a 96-well plate with optical bottom at 6700 cells/well, in 100 μl of complete culture medium. Twenty-four hours after, freshly completed medium containing several concentrations of HMPSNE or AOAA were added to the cells and the plates were incubated for 48 h at 37 °C and 5% CO_2_. After incubation we washed the cells twice with HBSS buffer (Gibco) and added 100 μM of AzMC diluted in HBSS buffer. The cells were then incubated 1 h at 37 °C and 5% CO_2_. The pictures were taken with a fluorescent microscope (Leica DFC360FX, 10x objective) and the ratio AzMC fluorescence intensity per cell was quantified by ImageJ software (NIH, Bethesda, MD, USA).

**Western blotting.** The cells were scraped from the well plate surface and lysed with RIPA lysis buffer (Thermo Fisher Scientific) supplemented with Halt™ Protease and Phosphatase Inhibitor Cocktail (Thermo Fisher Scientific) and sonicated 15s in an ultrasonic bath (XUBA3, Grant, United Kingdom). The protein concentrations, gel electrophoresis, proteins transfer, antibodies incubations, blot development and quantification were performed as described in Ref. [20]. Intensity values of related bands were normalized to β-actin housekeeping protein values. Representative blots of at least four independent experiments are shown.

**Quantitative real-time PCR (qRT-PCR).** As previously described in Ref. [20], the RNA was isolated using NucleoSpin® RNA Plus kit (Macherey-Nagel, Dueren, Germany) and quantified with a NanoDrop™ spectrophotometer (Thermo Fisher Scientific). The cDNA was synthesized from 1 μg total RNA with PrimeScript RT reagent kit (Takara, Shimogyō-ku, Kyoto, Japan) in the presence of random primers. The qPCRs were performed using SensiFAST SYBR Hi-ROX Kit (Meridian Bioscience, Cincinnati, OH, USA) on a StepOne Plus Real-Time PCR System (Applied Biosystems, Waltham, MA, USA). Each assay was performed in duplicates for each sample, and the GAPDH expression was used as an internal control. The relative expression ratio was calculated using the 2-ΔΔCt method. Representative qRT-PCRs of at least four independent experiments are shown. The pairs of primers used were: S1PR2 – Fw 5'-GCG CAC CTG TCC TGT ACT C-3', Rev 5'-GTT GGT GAG CGT GTA GAT GAT G-3'; ATF1 – Fw 5'-AGG ACT CAT CCG ACA GCA TAG-3', Rev 5'-TTC TGC CCC GTG TAT CTT CAG-3'; CyR61 – Fw 5’-CTCGCCTTAGTCGTCACCC-3’, Rev 5’- CGCCGAAGTTGCATTCCAG-3’.

**Migration and Proliferation Assay**. As previously described in Ref. [20] briefly, in the migration assays HCT116 ShCTR and HCT116 ShCyR61 cells were plated at 50,000 cells/well in a 96-well plate and incubated 24 h at 37 °C and 5% CO_2_ to create a monolayer of cells. The WoundMaker from Essen BioScience (Ann Arbor, MI, USA) was used to create homogeneous wide wounds. In the proliferation assays HCT116 ShCTR and HCT116 ShCyR61 cells were seeded in a 96-well plate at 13,400 cells/well. The cells from the migration and proliferation assays were then incubated in IncuCyte device (10× objective) (Essen Bioscience) and the confluence was recorded every 2 h by phase/contrast scanning for 48 h at 37 °C and 5% CO_2_. Images of at least four independent experiments were analyzed using the IncuCyte ZOOM 2018A software (Essen Bioscience).

## Author contributions

**KA**: experimental design, experimentation, data collection, statistical analysis, data interpretation, figure preparation, manuscript writing; **BL**: experimentation, data collection; **CS**: acquiring grant support, supervision, experimental design, data interpretation, manuscript writing.

## Declaration of competing interest

None.

## Data Availability

Data will be made available on request.

## **Annexin V Assay.** HCT116 cells were seeded in a 96-well plate at 6700 cells/well, HT-29 and LoVo cells at 13,400 cells/well in 100 μl of complete culture medium. Twenty-four hours after, freshly completed medium containing several concentrations of HMPSNE was added to the cells and the plates were incubated for 48 h at 37 °C and 5% CO_2_. After incubation the cells were detached and washed twice with cold BioLegend Cell Staining Buffer (Biolegend, San Diego, CA, USA), then resuspended in Annexin V Binding Buffer (Biolegend) containing an optimal dilution of APC Annexin V (Biolegend) and Propidium Iodide Solution (PI, Biolegend) and incubated 15 min at RT, in the dark. The cells were then analyzed using a FACS Fortessa flow cytometer (BD Biosciences, Franklin Lakes, NJ, USA). Emission signals for APC (Red 670/30) and PI (Blue 610/20) were detected. Data were analyzed with FlowJo (v10.8.1) software. Data shown are representative of FACS from at least four independent experiments in triplicates

**Vectors amplification.** As previously described in Ref. [20], the CyR61 promoter vector (HPRM43750-LvPM02) and the negative control (NEG-LvPM02) were obtained from GeneCopoeia (Rockville, MD, USA). The vectors were amplified, and their integrity were confirmed by digestion with BglII and MluI enzymes (New England BioLabs Inc. (NEB), Ipswich, MA, USA). The shERWOOD UltramiR Lentiviral shRNA target gene set for gene CYR61 (pZIP-hCMV-Puromycin-V204-1_Sh1 CyR61_ULTRA-3308242; pZIP-hCMV-Puromycin-V204-1_Sh2 CyR61_ULTRA-3308246; pZIP-hCMV-Puromycin-V204-1_Sh3 CyR61_ULTRA-3308247) used to perform the CyR61 silencing and the negative control (pZIP-hCMV-Puromycin-V204-1_ShCTR_ULTRA-NT#4_TLNSU4433) were obtained from Transomic Technologies, Inc (Huntsville, AL, USA). The vectors were amplified, and their integrity were confirmed by digestion with SalI and NcoI enzymes (NEB).

**Lentiviral production and HCT116 transduction.** As previously described in Ref. [20], viral particles were produced in HEK293T cells (ATCC) using third generation lentiviral system. Lentiviral supernatants were collected after 24 h and filtered via a 0.45 μm filtration unit and subsequently aliquoted and stored at −80 °C until use. Human HCT116 cells were transduced with lentiviral supernatant in the presence of 6 μg/ml protamine sulfate. Seventy-two hours following transduction, puromycin (1 μg/ml) was added to the culture to select transduced cells.

**CyR61 Promoter Assay.** As previously described in Ref. [20], HCT116 cells were seeded one day before the assay in 96-well plates. Complete medium containing GYY4137 or Na_2_S_3_ serial dilutions was added to the cells followed by an incubation of 48 h at 37 °C and 5% CO_2_. The cells were then detached and incubated in an optimal dilution of a LIVE/DEAD™ Fixable Near-IR Dead Cell Stain Kit (Invitrogen, Thermo Fisher Scientific) for 15 min, on ice. The cells were then analyzed using a FACS Fortessa flow cytometer (BD Biosciences). Emission signals for mCherry (610/20) and Live/dead IR (780/60) were detected. Data were analyzed with FlowJo (v10.8.1) software. Data shown are representative of FACS from at least four independent experiments in triplicates.

**ELISA.** The secreted CyR61 proteins were quantified using a Human CYR61/CCN1 ELISA Kit (Abcam, ab213772) according to manufacturer’s instructions. Data shown are representatives of ELISAs from at least four independent experiments in duplicates.

**Statistical Analysis.** Data are shown as mean ± SEM. One-way ANOVA with Dunnett’s multiple comparisons test, Kruskal-Wallis with Dunn’s multiple comparisons test, two-way ANOVA Sidak's multiple comparisons test and unpaired *t*-test were used to detect differences between groups. Statistically significant differences between these two groups are indicated by *p<0.05 or **p<0.01. Statistical calculations were performed using Graphpad Prism analysis software.

## References

[1] Szabo C., Papapetropoulos A. (2017). International union of basic and clinical pharmacology. CII: pharmacological modulation of H_2_S levels: H_2_S donors and H_2_S biosynthesis inhibitors. Pharmacol. Rev..

[2] Szabo C. (2018). A timeline of hydrogen sulfide (H_2_S) research: from environmental toxin to biological mediator. Biochem. Pharmacol..

[3] Cirino G., Szabo C., Papapetropoulos A. (2022 Apr 18). Physiological roles of hydrogen sulfide in mammalian cells, tissues and organs. Physiol. Rev..

[4] Zuhra K., Tome C.S., Forte E., Vicente J.B., Giuffre A. (2021). The multifaceted roles of sulfane sulfur species in cancer-associated processes. Biochim. Biophys. Acta Bioenerg..

[5] Szabo C., Coletta C., Chao C., Modis K., Szczesny B., Papapetropoulos A., Hellmich M.R. (2013). Tumor-derived hydrogen sulfide, produced by cystathionine-beta-synthase, stimulates bioenergetics, cell proliferation, and angiogenesis in colon cancer. Proc. Natl. Acad. Sci. USA.

[6] Bhattacharyya S., Saha S., Giri K., Lanza I.R., Nair K.S., Jennings N.B., Rodriguez-Aguayo C., Lopez-Berestein G., Basal E., Weaver A.L., Visscher D.W., Cliby W., Sood A.K., Bhattacharya R., Mukherjee P. (2013). Cystathionine beta-synthase (CBS) contributes to advanced ovarian cancer progression and drug resistance. PLoS One.

[7] Modis K., Coletta C., Asimakopoulou A., Szczesny B., Chao C., Papapetropoulos A., Hellmich M.R., Szabo C. (2014). Effect of S-adenosyl-L-methionine (SAM), an allosteric activator of cystathionine-beta-synthase (CBS) on colorectal cancer cell proliferation and bioenergetics in vitro. Nitric Oxide.

[8] Szabo C. (2016). Gasotransmitters in cancer: from pathophysiology to experimental therapy. Nat. Rev. Drug Discov..

[9] Phillips C.M., Zatarain J.R., Nicholls M.E., Porter C., Widen S.G., Thanki K., Johnson P., Jawad M.U., Moyer M.P., Randall J.W., Hellmich J.L., Maskey M., Qiu S., Wood T.G., Druzhyna N., Szczesny B., Modis K., Szabo C., Chao C., Hellmich M.R. (2017). Upregulation of cystathionine-beta-synthase in colonic epithelia reprograms metabolism and promotes carcinogenesis. Cancer Res..

[10] Untereiner A.A., Pavlidou A., Druzhyna N., Papapetropoulos A., Hellmich M.R., Szabo C. (2018). Drug resistance induces the upregulation of H_2_S-producing enzymes in HCT116 colon cancer cells. Biochem. Pharmacol..

[11] Olah G., Modis K., Toro G., Hellmich M.R., Szczesny B., Szabo C. (2018). Role of endogenous and exogenous nitric oxide, carbon monoxide and hydrogen sulfide in HCT116 colon cancer cell proliferation. Biochem. Pharmacol..

[12] Panza E., De Cicco P., Armogida C., Scognamiglio G., Gigantino V., Botti G., Germano D., Napolitano M., Papapetropoulos A., Bucci M., Cirino G., Ianaro A. (2015). Role of the cystathionine gamma lyase/hydrogen sulfide pathway in human melanoma progression. Pigment Cell. Melanoma Res..

[13] Wahafu W., Gai J., Song L., Ping H., Wang M., Yang F., Niu Y., Xing N. (2018). Increased H_2_S and its synthases in urothelial cell carcinoma of the bladder, and enhanced cisplatin-induced apoptosis following H_2_S inhibition in EJ cells. Oncol. Lett..

[14] Augsburger F., Szabo C. (2020). Potential role of the 3-mercaptopyruvate sulfurtransferase (3-MST)-hydrogen sulfide (H_2_S) pathway in cancer cells. Pharmacol. Res..

[15] Erdélyi K., Ditrói T., Johansson H.J., Czikora Á., Balog N., Silwal-Pandit L., Ida T., Olasz J., Hajdú D., Mátrai Z., Csuka O., Uchida K., Tóvári J., Engebraten O., Akaike T., Børresen Dale A.L., Kásler M., Lehtiö J., Nagy P. (2021). Reprogrammed transsulfuration promotes basal-like breast tumor progression via realigning cellular cysteine persulfidation. Proc. Natl. Acad. Sci. USA.

[16] Asimakopoulou A., Panopoulos P., Chasapis C.T., Coletta C., Zhou Z.M., Cirino G., Giannis A., Szabo C., Spyroulias G.A., Papapetropoulos A. (2013). Selectivity of commonly used pharmacological inhibitors for cystathionine synthase (CBS) and cystathionine lyase (CSE), Brit. J. Pharmacol..

[17] Zuhra K., Augsburger F., Majtan T., Szabo C. (2020). Cystathionine-beta-synthase: molecular regulation and pharmacological inhibition. Biomolecules.

[18] Hanaoka K., Sasakura K., Suwanai Y., Toma-Fukai S., Shimamoto K., Takano Y., Shibuya N., Terai T., Komatsu T., Ueno T., Ogasawara Y., Tsuchiya Y., Watanabe Y., Kimura H., Wang C., Uchiyama M., Kojima H., Okabe T., Urano Y., Shimizu T., Nagano T. (2017). Discovery and mechanistic characterization of selective inhibitors of H_2_S-producing enzyme: 3-mercaptopyruvate sulfurtransferase (3MST) targeting active-site cysteine persulfide. Sci. Rep..

[19] Augsburger F., Randi E.B., Jendly M., Ascencao K., Dilek N., Szabo C. (2020). Role of 3-mercaptopyruvate sulfurtransferase in the regulation of proliferation, migration, and bioenergetics in murine colon cancer cells. Biomolecules.

[20] Ascencao K., Dilek N., Augsburger F., Panagaki T., Zuhra K., Szabo C. (2021). Pharmacological induction of mesenchymal-epithelial transition via inhibition of H_2_S biosynthesis and consequent suppression of ACLY activity in colon cancer cells. Pharmacol. Res..

[21] Bantzi M., Augsburger F., Loup J., Berset Y., Vasilakaki S., Myrianthopoulos V., Mikros E., Szabo C., Bochet C.G. (2021). Novel aryl-substituted pyrimidones as inhibitors of 3-mercaptopyruvate sulfurtransferase with antiproliferative efficacy in colon cancer. J. Med. Chem..

[22] Chao C., Zatarain J.R., Ding Y., Coletta C., Mrazek A.A., Druzhyna N., Johnson P., Chen H.Y., Hellmich J.L., Asimakopoulou A., Yanagi K., Olah G., Szoleczky P., Toro G., Bohanon F.J., Cheema M., Lewis R., Eckelbarger D., Ahmad A., Modis K., Untereiner A., Szczesny B., Papapetropoulos A., Zhou J., Hellmich M.R., Szabo C. (2016). Cystathionine-beta-synthase inhibition for colon cancer: enhancement of the efficacy of aminooxyacetic acid via the prodrug approach. Mol. Med..

[23] Szczesny B., Marcatti M., Zatarain J.R., Druzhyna N., Wiktorowicz J.E., Nagy P., Hellmich M.R., Szabo C. (2016). Inhibition of hydrogen sulfide biosynthesis sensitizes lung adenocarcinoma to chemotherapeutic drugs by inhibiting mitochondrial DNA repair and suppressing cellular bioenergetics. Sci. Rep..

[24] Yue T., Zuo S., Bu D., Zhu J., Chen S., Ma Y., Ma J., Guo S., Wen L., Zhang X., Hu J., Wang Y., Yao Z., Chen G., Wang X., Pan Y., Wang P., Liu Y. (2020). Aminooxyacetic acid (AOAA) sensitizes colon cancer cells to oxaliplatin via exaggerating apoptosis induced by ROS. J. Cancer.

[25] Ye F., Li X., Sun K., Xu W., Shi H., Bian J., Lu R., Ye Y. (2020). Inhibition of endogenous hydrogen sulfide biosynthesis enhances the anti-cancer effect of 3,3'-diindolylmethane in human gastric cancer cells. Life Sci..

[26] Huang X., Xiang L., Li Y., Zhao Y., Zhu H., Xiao Y., Liu M., Wu X., Wang Z., Jiang P., Qing H., Zhang Q., Liu G., Zhang W., Li A., Chen Y., Liu S., Wang J. (2018). Snail/FOXK1/Cyr61 signaling axis regulates the epithelial-mesenchymal transition and metastasis in colorectal cancer. Cell. Physiol. Biochem..

[27] Yan J., Yang B., Lin S., Xing R., Lu Y. (2019). Downregulation of miR-142-5p promotes tumor metastasis through directly regulating CYR61 expression in gastric cancer. Gastric Cancer.

[28] Tanaka F., Rizqiawan A., Higashikawa K., Tobiume K., Okui G., Shigeishi H., Ono S., Shimasue H., Kamata N. (2013). Snail promotes Cyr61 secretion to prime collective cell migration and form invasive tumor nests in squamous cell carcinoma. Cancer Lett..

[29] Xie D., Miller C.W., O'Kelly J., Nakachi K., Sakashita A., Said J.W., Gornbein J., Koeffler H.P. (2001). Breast cancer - cyr61 is overexpressed, estrogen-inducible, and associated with more advanced disease. J. Biol. Chem..

[30] Sun Z.J., Wang Y., Cai Z., Chen P.P., Tong X.J., Xie D. (2008). Involvement of Cyr61 in growth, migration, and metastasis of prostate cancer cells. Br. J. Cancer.

[31] Kassis J.N., Virador V.M., Guancial E.A., Kimm D., Ho A.S., Mishra M., Chuang E.Y., Cook J., Gius D., Kohn E.C. (2009). Genomic and phenotypic analysis reveals a key role for CCN1 (CYR61) in BAG3-modulated adhesion and invasion. J. Pathol..

[32] Tsai M.S., Bogart D.F., Castaneda J.M., Li P., Lupu R. (2002). Cyr61 promotes breast tumorigenesis and cancer progression. Oncogene.

[33] Gery S., Xie D., Yin D., Gabra H., Miller C., Wang H., Scott D., Yi W.S., Popoviciu M.L., Said J.W., Koeffler H.P. (2005). Ovarian carcinomas: CCN genes are aberrantly expressed and CCN1 promotes proliferation of these cells. Clin. Cancer Res..

[34] Babic A.M., Kireeva M.L., Kolesnikova T.V., Lau L.F. (1998). CYR61, a product of a growth factor-inducible immediate early gene, promotes angiogenesis and tumor growth. Proc. Natl. Acad. Sci. USA.

[35] Lin M.T., Zuon C.Y., Chang C.C., Chen S.T., Chen C.P., Lin B.R., Wang M.Y., Jeng Y.M., Chang K.J., Lee P.H., Chen W.J., Kuo M.L. (2005). Cyr61 induces gastric cancer cell motility/invasion via activation of the integrin/nuclear factor-kappaB/cyclooxygenase-2 signaling pathway. Clin. Cancer Res..

[36] Xie D., Yin D., Wang H.J., Liu G.T., Elashoff R., Black K., Koeffler H.P. (2004). Levels of expression of CYR61 and CTGF are prognostic for tumor progression and survival of individuals with gliomas. Clin. Cancer Res..

[37] Wang G., Gu J., Gao Y. (2016). MicroRNA target for MACC1 and CYR61 to inhibit tumor growth in mice with colorectal cancer. Tumour Biol..

[38] Li J., Ye L., Owen S., Weeks H.P., Zhang Z., Jiang W.G. (2015). Emerging role of CCN family proteins in tumorigenesis and cancer metastasis (Review). Int. J. Mol. Med..

[39] Lau L.F. (2011). CCN1/CYR61: the very model of a modern matricellular protein. Cell. Mol. Life Sci..

[40] Xie L., Song X., Lin H., Chen Z., Li Q., Guo T., Xu T., Su T., Xu M., Chang X., Wang L.K., Liang B., Huang D. (2019). Aberrant activation of CYR61 enhancers in colorectal cancer development. J. Exp. Clin. Cancer Res..

[41] Jeong D., Heo S., Sung Ahn T., Lee S., Park S., Kim H., Park D., Byung Bae S., Lee S.S., Soo Lee M., Kim C.J., Jun Baek M. (2014). Cyr61 expression is associated with prognosis in patients with colorectal cancer. BMC Cancer.

[42] Ladwa R., Pringle H., Kumar R., West K. (2011). Expression of CTGF and Cyr61 in colorectal cancer. J. Clin. Pathol..

[43] Baek M., Bae S., Jeong D. (2011). Relationship of pro-angiogenic factor Cyr61 to colorectal cancer development and prognosis. J. Clin. Oncol..

[44] Monnier Y., Farmer P., Bieler G., Imaizumi N., Sengstag T., Alghisi G.C., Stehle J.C., Ciarloni L., Andrejevic-Blant S., Moeckli R., Mirimanoff R.O., Goodman S.L., Delorenzi M., Ruegg C. (2008). CYR61 and alphaVbeta5 integrin cooperate to promote invasion and metastasis of tumors growing in preirradiated stroma. Cancer Res..

[45] Han J.S., Macarak E., Rosenbloom J., Chung K.C., Chaqour B. (2003). Regulation of Cyr61/CCN1 gene expression through RhoA GTPase and p38MAPK signaling pathways. Eur. J. Biochem..

[46] You J.J., Yang C.M., Chen M.S., Yang C.H. (2010). Regulation of Cyr61/CCN1 expression by hypoxia through cooperation of c-Jun/AP-1 and HIF-1alpha in retinal vascular endothelial cells. Exp. Eye Res..

[47] Karin M., Liu Z., Zandi E. (1997). AP-1 function and regulation. Curr. Opin. Cell Biol..

[48] Young N., Van Brocklyn J.R. (2007). Roles of sphingosine-1-phosphate (S1P) receptors in malignant behavior of glioma cells. Differential effects of S1P2 on cell migration and invasiveness. Exp. Cell Res..

[49] Feng Z.P., Deng H.C., Jiang R., Du J., Cheng D.Y. (2015). Involvement of AP-1 in p38MAPK signaling pathway in osteoblast apoptosis induced by high glucose. Genet. Mol. Res..

[50] Basu S., Haase G., Ben-Ze'ev A. (2016). Wnt signaling in cancer stem cells and colon cancer metastasis. F1000Res.

[51] Schatoff E.M., Leach B.I., Dow L.E. (2017). Wnt signaling and colorectal cancer. Curr. Colorectal Cancer Rep..

[52] Xie D., Yin D., Tong X.J., O'Kelly J., Mori A., Miller C., Black K., Gui D., Said J.W., Koeffler H.P. (2004). Cyr61 is overexpressed in gliomas and involved in integrin-linked kinase-mediated Akt and beta-catenin-TCF/Lef signaling pathways. Cancer Res..

[53] Li Z.Q., Ding W., Sun S.J., Li J., Pan J., Zhao C., Wu W.R., Si W.K. (2012). Cyr61/CCN1 is regulated by Wnt/beta-catenin signaling and plays an important role in the progression of hepatocellular carcinoma. PLoS One.

[54] Sano M., Driscoll D.R., DeJesus-Monge W.E., Quattrochi B., Appleman V.A., Ou J., Zhu L.J., Yoshida N., Yamazaki S., Takayama T., Sugitani M., Nemoto N., Klimstra D.S., Lewis B.C. (2016). Activation of WNT/beta-catenin signaling enhances pancreatic cancer development and the malignant potential via up-regulation of Cyr61. Neoplasia.

[55] Guo Z.Y., Hao X.H., Tan F.F., Pei X., Shang L.M., Jiang X.L., Yang F. (2011). The elements of human cyclin D1 promoter and regulation involved. Clin. Epigenet..

[56] Montalto F.I., De Amicis F. (2020). Cyclin D1 in cancer: a molecular connection for cell cycle control, adhesion and invasion in tumor and stroma. Cells.

[57] Fox K.E., Colton L.A., Erickson P.F., Friedman J.E., Cha H.C., Keller P., MacDougald O.A., Klemm D.J. (2008). Regulation of cyclin D1 and Wnt10b gene expression by cAMP-responsive element-binding protein during early adipogenesis involves differential promoter methylation. J. Biol. Chem..

[58] Alao J.P. (2007). The regulation of cyclin D1 degradation: roles in cancer development and the potential for therapeutic invention. Mol. Cancer.

[59] Masamha C.P., Benbrook D.M. (2009). Cyclin D1 degradation is sufficient to induce G(1) cell cycle arrest despite constitutive expression of cyclin E2 in ovarian cancer cells. Cancer Res..

[60] Radu A., Neubauer V., Akagi T., Hanafusa H., Georgescu M.M. (2003). PTEN induces cell cycle arrest by decreasing the level and nuclear localization of cyclin D1. Mol. Cell Biol..

[61] O'Connor M.J., Thakar T., Nicolae C.M., Moldovan G.L. (2021). PARP14 regulates cyclin D1 expression to promote cell-cycle progression. Oncogene.

[62] Xiao Y., Wang J., Lu J., Liu Y., Wang Y., Gao Y., Jin D. (2011). Down-regulation of cyclin D1 by small interfering RNA inhibits cell growth and induces apoptosis of laryngeal squamous cell carcinoma. Am. J. Otolaryngol..

[63] Liang Y., Li C., Guzman V.M., 3rd Evinger A.J., Protzman C.E., Krauss A.H., Woodward D.F. (2003). Comparison of prostaglandin F2alpha, bimatoprost (prostamide), and butaprost (EP2 agonist) on Cyr61 and connective tissue growth factor gene expression. J. Biol. Chem..

[64] Zhang K., Jiang D. (2017). RhoA inhibits the hypoxia-induced apoptosis and mitochondrial dysfunction in chondrocytes via positively regulating the CREB phosphorylation. Biosci. Rep..

[65] Goncharenko-Khaider N., Lane D., Matte I., Rancourt C., Piche A. (2010). The inhibition of Bid expression by Akt leads to resistance to TRAIL-induced apoptosis in ovarian cancer cells. Oncogene.

[66] Jin Y., Kim H.P., Cao J., Zhang M., Ifedigbo E., Choi A.M. (2009). Caveolin-1 regulates the secretion and cytoprotection of Cyr61 in hyperoxic cell death. Faseb. J..

[67] Harris L.G., Pannell L.K., Singh S., Samant R.S., Shevde L.A. (2012). Increased vascularity and spontaneous metastasis of breast cancer by hedgehog signaling mediated upregulation of cyr61. Oncogene.

[68] Maity G., Mehta S., Haque I., Dhar K., Sarkar S., Banerjee S.K., Banerjee S. (2014). Pancreatic tumor cell secreted CCN1/Cyr61 promotes endothelial cell migration and aberrant neovascularization. Sci. Rep..

[69] Filipovic M.R. (2015). Persulfidation (S-sulfhydration) and H_2_S. Handb. Exp. Pharmacol..

[70] Nagy P. (2015). Mechanistic chemical perspective of hydrogen sulfide signaling. Methods Enzymol..

[71] Zhang D., Du J., Tang C., Huang Y., Jin H. (2017). H_2_S-induced sulfhydration: biological function and detection methodology. Front. Pharmacol..

[72] Kimura H. (2021). Hydrogen sulfide (H_2_S) and polysulfide (H_2_S_n_) signaling: the first 25 years. Biomolecules.

[73] Greiner R., Pálinkás Z., Bäsell K., Becher D., Antelmann H., Nagy P., Dick T.P. (2013). Polysulfides link H_2_S to protein thiol oxidation. Antioxidants Redox Signal..

[74] Bátai I.Z., Sár C.P., Horváth Á., Borbély É., Bölcskei K., Kemény Á., Sándor Z., Nemes B., Helyes Z., Perkecz A., Mócsai A., Pozsgai G., Pintér E. (2019). TRPA1 ion channel determines beneficial and detrimental effects of GYY4137 in murine serum-transfer arthritis. Front. Pharmacol..

[75] Xie L., Gu Y., Wen M., Zhao S., Wang W., Ma Y., Meng G., Han Y., Wang Y., Liu G., Moore P.K., Wang X., Wang H., Zhang Z., Yu Y., Ferro A., Huang Z., Ji Y. (2016). Hydrogen sulfide induces Keap1 S-sulfhydration and suppresses diabetes-accelerated atherosclerosis via Nrf2 activation. Diabetes.

[76] Sen N., Paul B.D., Gadalla M.M., Mustafa A.K., Sen T., Xu R., Kim S., Snyder S.H. (2012). Hydrogen sulfide-linked sulfhydration of NF-kappaB mediates its antiapoptotic actions. Mol. Cell..

[77] Zhang D., Wang X., Chen S., Chen S., Yu W., Liu X., Yang G., Tao Y., Tang X., Bu D., Zhang H., Kong W., Tang C., Huang Y., Du J., Jin H. (2019). Endogenous hydrogen sulfide sulfhydrates IKKbeta at cysteine 179 to control pulmonary artery endothelial cell inflammation. Clin. Sci. (Lond.).

[78] Saha S., Chakraborty P.K., Xiong X., Dwivedi S.K., Mustafi S.B., Leigh N.R., Ramchandran R., Mukherjee P., Bhattacharya R. (2016). Cystathionine beta-synthase regulates endothelial function via protein S-sulfhydration. Faseb. J..

[79] Meng G., Xiao Y., Ma Y., Tang X., Xie L., Liu J., Gu Y., Yu Y., Park C.M., Xian M., Wang X., Ferro A., Wang R., Moore P.K., Zhang Z., Wang H., Han Y., Ji Y. (2016). Hydrogen sulfide regulates Krüppel-Like Factor 5 transcription activity via specificity protein 1 S-sulfhydration at Cys664 to prevent myocardial hypertrophy. J. Am. Heart Assoc..

[80] Dou Q., Hao F., Sun L., Xu X., Cui M.Z. (2017). CRE and SRE mediate LPA-induced CCN1 transcription in mouse aortic smooth muscle cells, Can. J. Physiol. Pharmacol..

[81] Pedre B., Dick T.P. (2020). 3-Mercaptopyruvate sulfurtransferase: an enzyme at the crossroads of sulfane sulfur trafficking. Biol. Chem..

[82] Grote K., Salguero G., Ballmaier M., Dangers M., Drexler H., Schieffer B. (2007). The angiogenic factor CCN1 promotes adhesion and migration of circulating CD34+ progenitor cells: potential role in angiogenesis and endothelial regeneration. Blood.

[83] Yu Y., Gao Y., Wang H., Huang L., Qin J., Guo R., Song M., Yu S., Chen J., Cui B., Gao P. (2008). The matrix protein CCN1 (CYR61) promotes proliferation, migration and tube formation of endothelial progenitor cells. Exp. Cell Res..

[84] Gao L., Fan Y., Hao Y., Yuan P., Liu D., Jing Z., Zhang Z. (2017). Cysteine-rich 61 (Cyr61) upregulated in pulmonary arterial hypertension promotes the proliferation of pulmonary artery smooth muscle cells. Int. J. Med. Sci..

[85] Zhang H., Lian M., Zhang J., Bian Z., Tang R., Miao Q., Peng Y., Fang J., You Z., Invernizzi P., Wang Q., Gershwin M.E., Ma X. (2018). A functional characteristic of cysteine-rich protein 61: modulation of myeloid-derived suppressor cells in liver inflammation. Hepatology.

[86] Jun J.I., Lau L.F. (2020). CCN1 is an opsonin for bacterial clearance and a direct activator of toll-like receptor signaling. Nat. Commun..

[87] Kim H., Son S., Ko Y., Lee J.E., Kim S., Shin I., YAP (2021). CTGF and Cyr61 are overexpressed in tamoxifen-resistant breast cancer and induce transcriptional repression of ERalpha. J. Cell Sci..

[88] Maity G., Ghosh A., Gupta V., Haque I., Sarkar S., Das A., Dhar K., Bhavanasi S., Gunewardena S.S., Von Hoff D.D., Mallik S., Kambhampati S., Banerjee S.K., Banerjee S. (2019). CYR61/CCN1 regulates dCK and CTGF and causes gemcitabine-resistant phenotype in pancreatic ductal adenocarcinoma. Mol. Cancer Therapeut..

[89] Song Y., Kang Y., Lin Z., Zeng M., Shi P., Lin J., Lu P., Luo L., Cao Y., Zhu X. (2021). Cyr61 mediates oxaliplatin resistance in colorectal cancer cells by regulating Bcl-xL expression. J. Cancer.

[90] Espinoza I., Lupu R. (2009). CYR61 expression promotes taxol resistance: a molecular link with the p53 in breast. Cancer Res..

[91] Kitagawa K., Shigemura K., Sung S.Y., Chen K.C., Huang C.C., Chiang Y.T., Liu M.C., Huang T.W., Yamamichi F., Shirakawa T., Fujisawa M. (2019). Possible correlation of sonic hedgehog signaling with epithelial-mesenchymal transition in muscle-invasive bladder cancer progression. J. Cancer Res. Clin. Oncol..

[92] Xie Z., Wang F., Lin L., Duan S., Liu X., Li X., Li T., Xue M., Cheng Y., Ren H., Zhu Y. (2020). An SGLT2 inhibitor modulates SHH expression by activating AMPK to inhibit the migration and induce the apoptosis of cervical carcinoma cells. Cancer Lett..

[93] Zhao G., Li H., Guo Q., Zhou A., Wang X., Li P., Zhang S. (2020). Exosomal sonic hedgehog derived from cancer-associated fibroblasts promotes proliferation and migration of esophageal squamous cell carcinoma. Cancer Med..

[94] Szczesny B., Módis K., Yanagi K., Coletta C., Le Trionnaire S., Perry A., Wood M.E., Whiteman M., Szabo C. (2014). AP39, a novel mitochondria-targeted hydrogen sulfide donor, stimulates cellular bioenergetics, exerts cytoprotective effects and protects against the loss of mitochondrial DNA integrity in oxidatively stressed endothelial cells in vitro. Nitric Oxide.

